# Does Ablation Technique Utilized in the Management of Unresectable Locally Advanced Pancreatic Adenocarcinoma?

**DOI:** 10.3390/cancers2042098

**Published:** 2010-12-10

**Authors:** Raffaele Pezzilli, Dario Fabbri, Andrea Imbrogno

**Affiliations:** Department of Internal Medicine and Gastroenterology, S. Orsola-Malpighi Hospital, University of Bologna, Via Massarenti.9 40138, Bologna, Italy; E-Mails: dario.fabbri@gmail.com (D.F.); andrea_imbrogno@yahoo.com (A.I.)

**Keywords:** pancreatic neoplasms, catheter ablation, surgery

## Abstract

Radiofrequency ablation in the management of advanced pancreatic cancer should be no longer utilized in patients with locally advanced or metastatic pancreatic adenocarcinoma.

We have read with great interest the commentary of Dr. Spiliotis [1] about our review article focusing on the poor results of ablation technique in patients with unresectable pancreatic adenocarcinoma [2]. Of course, we agree with Dr. Spiliotis that the only beneficial procedure determining an acceptable good long term survival in patients with pancreatic adenocarcinoma remains the R0 pancreatic resection [3]. We also agree and that the majority of patients at the time of the diagnosis have unresectable tumors due to locally advanced or metastatic disease. Finally, we agree that both chemotherapy and chemoradiation therapy confer symptomatic improvement in these patients. Speaking about radiofrequency ablation (RFA) in patients with unresectable pancreatic adenocarcinoma, we also agree with Dr. Spiliotis that the human pancreatic tissue requires dedicated electrodes, optimal thermal kinetic parameters, optimal duration of thermal application; however, in all published studies, including that of Girelli *et al.* [4], no standardization of RFA were made; furthermore, long term results of this technique are lacking. In our opinion, based on the results of the data present in literature, RFA is not suitable for curing the patients with unresectable locally advanced pancreatic adenocarcinoma [1]. This opinion is also reinforced by a recent published study of our group [4]. In this prospective study we enrolled a homogenous population having the same stage and similar location of the tumor; both the surgical approach and the thermal ablation parameters were standardized in all operated patients: the long term results remained disappointing [5]. Using the results of this study it is possible to answer to the questions posed by Dr. Spiliotis: the study was carried out in an experienced center in which we use routinely the RFA mainly for treating hepatic tumors; we enrolled a homogenous population having the same stage of the disease and a similar location of the tumor; we carried out the same surgical approach in all patients; we validated the technique not only for the feasibility and safety, but also for the long term results. The results unchanged: they remained disappointing at least in our experimental conditions.
